# Molecular insights into volatile organic compound sensing and signaling in plants

**DOI:** 10.1111/tpj.70789

**Published:** 2026-03-14

**Authors:** Matthew E. Bergman, Sun Hyun Chang, Benoît Boachon, Nitzan Shabek, Natalia Dudareva

**Affiliations:** ^1^ Department of Biochemistry Purdue University West Lafayette Indiana 47907 USA; ^2^ Purdue Center for Plant Biology Purdue University West Lafayette Indiana 47907 USA; ^3^ Department of Plant Biology, College of Biological Sciences University of California–Davis Davis California 95616 USA; ^4^ Université Jean Monnet Saint‐Etienne CNRS, LBVpam EMR 5003 Saint‐Etienne F‐42023 France; ^5^ Department of Horticulture and Landscape Architecture Purdue University West Lafayette Indiana 47907 USA

**Keywords:** volatile organic compounds, perception of volatiles, receptor‐ligand interactions, KAI2‐mediated signaling, plant VOC receptors

## Abstract

Plants interact with their surrounding environment through the perception of a vast and structurally diverse array of volatile organic compounds (VOCs); however, the molecular mechanisms involved remain mostly unknown. Despite the large number of VOCs emitted and perceived by plants, only a small number of phylogenetically distinct, but often structurally similar receptors and receptor‐like proteins have been identified and characterized to date. In this review, we summarize the current knowledge on plant VOC perception, with an emphasis on the receptors involved, including their structural characteristics and ligand specificities, as well as how distinct VOC signals can be translated into different downstream physiological responses. We further highlight the involvement of KARRIKIN INSENSITIVE 2 (KAI2)‐mediated signaling in the perception of volatile compounds and their derivatives, discussing its potential role in expanding the repertoire of plant VOC perception mechanisms.

## Significance Statement

Plants interpret important environmental cues through their perception of volatile organic compounds (VOCs), yet the molecular mechanisms enabling this process remain poorly understood. This review provides current insights into receptor structure, ligand specificity, and downstream signaling, while highlighting major knowledge gaps in our understanding of how plants detect and respond to diverse VOC signals.

## THE ROLE OF VOCs IN PLANT COMMUNICATION

Plants interact with their surrounding environment through a chemical language involving structurally diverse volatile organic compounds (VOCs) in addition to non‐volatile signaling chemicals (Baldwin, 2010; Karban, 2021; Kessler et al., 2023; Mandal et al., 2010; Ninkovic et al., 2021; Sharifi et al., 2022; Tarkowská & Strnad, 2018). VOCs are small molecules emitted by all kingdoms of life (Vlot & Rosenkranz, 2022) as well as by human activities and natural disasters such as forest fires. The low boiling points and low molecular weight of these compounds allow them to volatilize and diffuse through the atmosphere, while their lipophilic nature enables their uptake by plants through the cuticle and partitioning into membranes and/or lipid droplets (Bergman et al., 2025; Wang & Erb, 2022; Widhalm et al., 2023). Upon perception inside the cell, many compounds elicit signaling responses, some of which have dramatic impacts on plant physiology and development. Plants themselves also emit VOCs from all organs, with roles in facilitating interactions with animals and microbes, communicating with other plants or distinct organs/tissues within the same plant, and mediating responses to abiotic stress factors (Brosset & Blande, 2022; Ravelo‐Ortega et al., 2023; Rosenkranz et al., 2021). As such, VOCs emitted from plants and neighboring organisms facilitate a complex network of interactions and information exchange that is impacted by environmental factors and human disturbances (Bergman et al., 2025; Kessler et al., 2023; Pinto‐Zevallos & Blande, 2024; Waterman et al., 2024; Wilson et al., 2015).

One of the most prominent roles of VOCs in plant communication is their function in mediating defense against herbivory. Infested plants often emit herbivore‐induced plant volatiles that are perceived by neighboring plants and elicit defense priming (Aratani et al., 2023; Grandi et al., 2024; Rosenkranz et al., 2021; Schuman, 2023). In addition, some emitted VOCs can actively repel herbivores (Yu et al., 2024) or even signal parasitic insects to target the attacking herbivores, thus protecting plants via tritrophic interactions (Turlings & Erb, 2018; Zhang et al., 2025). While the emission of VOCs for defense is a generally effective strategy, some attackers, including insects and microorganisms, manipulate this communication by mimicking plant signals or suppressing defense signaling pathways (Erb & Reymond, 2019; Fu et al., 2025; Ji et al., 2021; Kessler, 2025; Lin et al., 2021; Yamasaki et al., 2021), resulting in a bidirectional ‘signaling arms race’ (Jones et al., 2021; Kessler, 2025). In general, plants are not only targets of plant‐emitted VOCs, but they also perceive volatile cues produced by microbes and herbivores, which may act either as positive or negative regulators of plant defenses (Bouwmeester et al., 2019; Schenkel et al., 2018). Emitted VOCs are released into the airspace where they create a diffuse chemical signal that is perceived by receiver plants. While the biosynthesis and, more recently, the emission of VOCs have been thoroughly studied (Liao et al., 2021, 2023; Ray et al., 2022; Widhalm et al., 2015), a detailed mechanistic understanding of plant VOC perception is still lacking.

## PERCEPTION OF VOCs

For plants to respond to volatile cues from the surrounding environment, the VOCs must first be taken up either directly through the cuticle or via stomata before they are perceived by receptors (Aguirre et al., 2023; Bergman et al., 2025; Maleki, Seidl‐Adams, Felton, et al., 2024; Matsui, 2016; Wang & Erb, 2022). To date, the relative contributions of the cuticle and stomatal routes to VOC uptake have not been quantified; however, the relative contribution of the cuticle likely varies based on its thickness and composition as well as the physicochemical properties of the individual VOC, similar to what is observed for emission (Aguirre et al., 2023; Bhattacharya & Mitra, 2023; Liao et al., 2021, 2023; Lin et al., 2022; Ray et al., 2022; Widhalm et al., 2023). Some compounds with relatively low Henry's law volatility constants may dissolve only sparingly into the cuticle and instead rely more on entry through the stomata (Aguirre et al., 2023; Bergman et al., 2025; Maleki, Seidl‐Adams, Felton, et al., 2024). Inside the plant, exogenous compounds can act at the site of entry and/or be transported through the vasculature to their destination (Maleki, Seidl‐Adams, Fahimi, et al., 2024; Tanaka et al., 2018; Zhang, Berman, & Shani, 2023).

Following initial uptake, VOCs could be transferred across the cell wall to the plasma membrane by extracellular non‐specific lipid transfer proteins (LTPs) (Arimura & Uemura, 2025; Liao et al., 2023; Widhalm et al., 2023). While no plasma membrane‐bound receptors are known for VOCs, it is possible that they could be perceived by unidentified dedicated odorant receptors or receptor‐like kinases (Arimura & Uemura, 2025; Fisher et al., 2025; Stratmann et al., 2025). Alternatively, these lipophilic VOCs can partition into the plasma membrane and/or extracellular vesicle membranes, or cross the plasma membrane via endocytosis, transporters or simple diffusion. Within the aqueous matrix of the cytosol, they can be modified by enzymes or directly interact with soluble or membrane anchored receptors. Intracellular VOC movement may be facilitated by LTPs or other small‐molecule carriers (Li et al., 2016; Melnikova et al., 2023), as well as lipid droplets, organelle interactions, and vesicle trafficking (Blot et al., 2025; Oikawa et al., 2019; Shimada et al., 2018), or possibly liquid–liquid phase separation (Li et al., 2025). Recent mathematical modeling that considered partition coefficients across cellular barriers showed that entry into the cell from the atmosphere by diffusion alone may be sufficient to reach intracellular concentrations within the expected range of soluble receptors' *K*_d_ values (Widhalm et al., 2023). Within the destination cells, whether originating extracellularly (e.g., airborne or synthesized elsewhere in the plant) or produced intracellularly, VOCs can directly bind to receptors that trigger downstream signaling cascades or alternatively be converted into less volatile but more potent signaling molecules or even into bioactive defense compounds (Sugimoto et al., 2023; Zhou et al., 2025). Some VOCs can also be metabolized through classical xenobiotic detoxification mechanisms such as glycosylation or conjugation to glutathione, possibly to prevent their toxic buildup within the cell (Matsui, 2016; Muramoto et al., 2015; Oikawa & Lerdau, 2013; Sugimoto et al., 2014, 2015).

**Figure 1:**
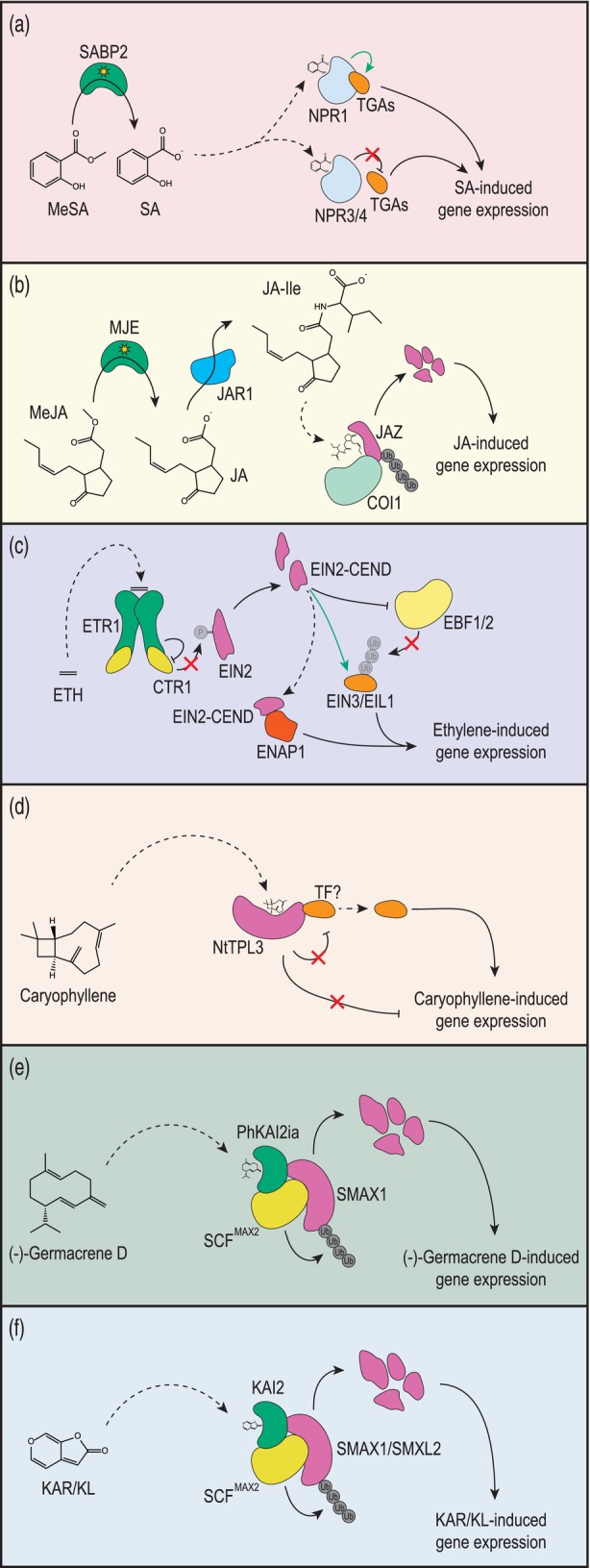
Simplified overview of plant volatile organic compound (VOC) perception. (a) Methyl salicylate (MeSA) is enzymatically demethylated to salicylate (SA) by the α/β hydrolase SALICYLIC ACID BINDING PROTEIN 2 (SABP2). Free SA then interacts with NONEXPRESSOR OF PATHOGENESIS‐RELATED GENES 1 (NPR1), a transcriptional co‐activator that associates with TGACG motif‐binding transcription factors (TGAs) to activate SA‐responsive gene expression. NPR3 and NPR4 negatively regulate TGA‐mediated transcription, partly through interactions with NPR1 and modulating its stability. The downstream consequences of SA signaling are concentration dependent and involve varied crosstalk with other signaling pathways, thus adding complexity beyond the simplified scheme presented here. For a detailed review of the signaling cascade, see (Spoel & Dong, 2024). (b) Methyl jasmonate (MeJA) is enzymatically demethylated to jasmonate (JA) by the α/β hydrolase MeJA esterase (MJE). JA is then conjugated to isoleucine by JASMONATE RESISTANT 1 (JAR1) to form (+)‐7‐iso‐jasmonoyl‐L‐isoleucine (JA‐Ile) which interacts with a co‐receptor complex composed of the F‐box protein CORONATINE INSENSITIVE1 (COI1) and the transcriptional co‐repressor JASMONATE‐ZIM‐DOMAIN (JAZ). Binding of JA‐Ile to the co‐receptor complex triggers ubiquitination and proteasomal degradation of JAZ, leading to JA‐induced transcriptional changes. (c) Ethylene is perceived by endoplasmic reticulum membrane‐bound ethylene receptors, such as ETHYLENE RESPONSE 1 (ETR1), which triggers inactivation of CONSTITUTIVE TRIPLE RESPONSE1 (CTR1). CTR1 inactivation prevents phosphorylation of ETHYLENE‐INSENSITIVE2 (EIN2) and allows it to be cleaved. The released C‐terminal domain of EIN2 (EIN2‐CEND) then indirectly activates EIN3 and EIN3‐LIKE1 (EIL1) transcription factors. EIN2‐CEND represses translation of EIN3‐Binding F‐box proteins (EBFs), which normally target EIN3 and EIL1 for proteasomal degradation. It also interacts with EIN2 NUCLEAR‐ASSOCIATED PROTEIN 1 (ENAP1) to promote histone acetylation and chromatin remodeling, facilitating transcription of ethylene‐responsive genes, partly driven by EIN3/EIL1. (d) The sesquiterpene β‐caryophyllene and its derivatives are perceived by transcriptional co‐repressors known as TOPLESS‐like proteins (TPLs) in *Nicotiana tabacum*, namely by NtTPL3. It is inferred that β‐caryophyllene binding to NtTPL3 relieves repression of currently unidentified transcription factor(s) (TF?) to enable β‐caryophyllene‐responsive gene expression. (e) In *Petunia hybrida* flowers, the sesquiterpene (−)‐germacrene D emitted from developing petal tubes is perceived in developing stigmas by PhKAI2ia, an intermediate clade KARRIKIN INSENSITIVE 2 (KAI2) receptor of the α/β hydrolase superfamily. Interaction of (−)‐germacrene D with PhKAI2ia triggers recruitment of the Skp, Cullin, F‐box (SCF) E3 ubiquitin ligase complex, including the F‐box protein MORE AXILLARY GROWTH2 (MAX2). SUPPRESSOR OF MAX2 1 (SMAX1) is then degraded, likely through ubiquitination and 26S proteasomal degradation, allowing (−)‐germacrene D‐induced changes in gene expression. (f) Karrikins (KARs) and/or KAI2 ligands (KLs) are perceived by KAI2 proteins, triggering recruitment of the SCF E3 ubiquitin ligase complex, with MAX2 as the F‐box protein. This leads to ubiquitination and 26S proteasomal degradation of the transcriptional co‐repressors SMAX1/SMAX1‐LIKE2 (SMXL2), enabling KAR/KL‐responsive gene expression changes. Solid black lines with arrows show processes, dashed black lines with arrows imply movement/translocation, solid black lines with blunt ends/crossbars show inhibition/repression, solid green lines with arrows represent activation, red cross through lines indicate blocked/inhibited processes. ‘Ub’ and ‘P’ in circles indicate ubiquitination and phosphorylation, respectively.

**Figure 2:**
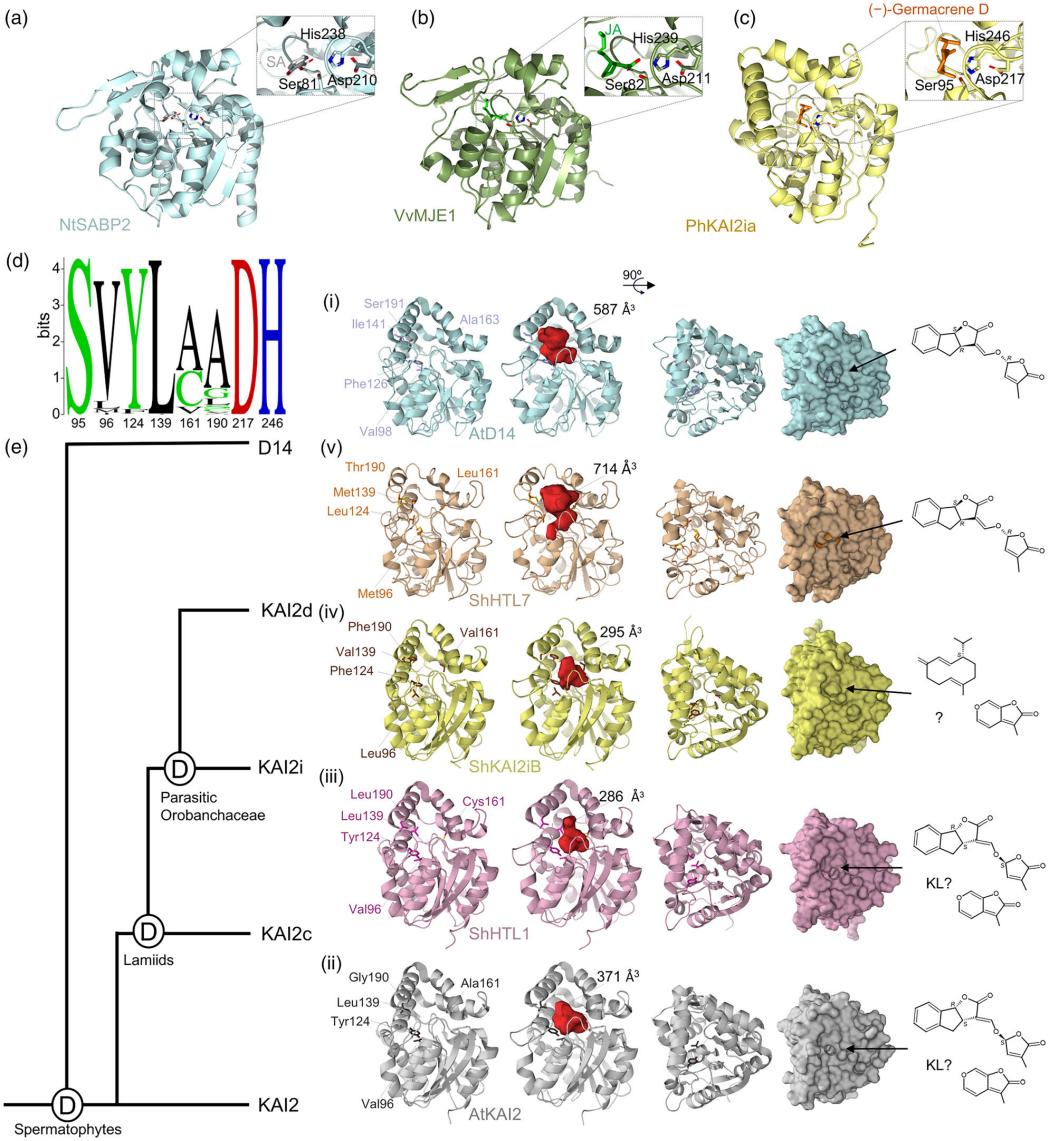
Structures of volatile molecule receptors and receptor‐like proteins in plants. (a) Crystal structure of *Nicotiana tabacum* SALICYLIC ACID BINDING PROTEIN 2 (NtSABP2; pale cyan) bound to salicylic acid (SA; light gray) with the sidechains shown for amino acid residues of the catalytic triad (PDB ID: 1Y7I); right, close‐up view of the catalytic pocket residues (Ser81, His238, Asp210) shown in stick representation. (b) Predicted structure of *Vitis vinifera* methyl jasmonate esterase 1/methylesterase 5 (VvMJE1; green) by AlphaFold3 followed by molecular docking simulation with jasmonic acid (JA; neon green) using Diffdock‐L (Corso et al., 2024); right, close‐up view of the catalytic pocket residues (Ser82, His239, Asp211) shown in stick representation. (c) Predicted structure of the intermediate clade KARRIKIN INSENSITIVE 2 (KAI2) that perceived (−)‐germacrene D in *Petunia hybrida* (PhKAI2ia; pale yellow) by AlphaFold2 with docked (−)‐germacrene D (orange) showing the catalytic triad; right, close‐up view of the catalytic pocket residues (Ser95, His246, Asp217) shown in stick representation. The structural model of PhKAI2ia was provided by Stirling et al. (2024). (d) KAI2 residue conservation shown as sequence logos for KAI2 sequences available in Phytozome v14 (Goodstein et al., 2012) aligned through clustal omega using Geneious Prime. Visualization was performed using WebLogo (Crooks et al., 2004). (e) Simplified phylogenetic tree of KAI2 clades in seed plants (left) and their representative crystal or predicted structures shown in side view, top view (ribbon), and surface representations with close‐up views (left to right). Key conserved residues that determine pocket shape (corresponding to Val96, Tyr124, Leu139, Ala161, and Gly190 of AtKAI2) are highlighted, ligand binding pockets are shown in red and cavity volumes are indicated. Schematics of the representative agonist molecules are shown at right. (i) DWARF14 (D14), the canonical strigolactone (SL) receptor, likely arose by duplication of KAI2 prior to the evolution of spermatophytes. The crystal structure of *Arabidopsis thaliana* AtD14 (pale cyan; PDB: 4IH4) serves as representative structure for D14 proteins. D14 perceives SL molecules with 2′R enantiomeric configuration (e.g., GR24^5DS^/(+)‐GR24) as agonists. (ii) The crystal structure of AtKAI2 (light gray; PDB ID: 4HRY) serves as a representative of the KAI2 clade. In spermatophytes, KAI2 functions as a receptor for KAI2 ligand(s) (KL) and/or karrikins (KARs) and perceives the synthetic agonist GR24^ent‐5DS^/(−)‐GR24, which possesses the 2′S configuration of the butanolide ring. (iii) In lamiids, KAI2 diverged into three clades. Conserved KAI2 (KAI2c) proteins, represented by the crystal structure of *Striga hermonthica* HYPOSENSITIVE TO LIGHT 1 (ShHTL1; light pink; PDB ID: 5Z7W), are highly conserved and hypothesized to be specific receptors for the endogenous KL. They are specifically sensitive to desmethyl butenolide analogs, such as desmethyl germinone A dMGer. (iv) The intermediate KAI2 (KAI2i) clade diverged through a gene duplication event from KAI2c followed by neofunctionalization. The KAI2i clade, represented by PhKAI2ia (pale yellow; structural model provided by Stirling et al. (2024)), is generally classified as being responsive to KARs despite some exceptions (e.g., perception of (−)‐germacreneD in PhKAI2ia). (v) KAI2d, represented by the crystal structure of ShHTL7 (beige; PDB ID: 5Z95) arose in the parasitic Orobanchaceae lineage via gene duplication and neofunctionalization. KAI2d proteins are predominantly characterized as specialized SL receptors, having evolved through convergent evolution to perceive host‐derived SLs and showing stronger responses to SLs possessing the 2′R enantiomeric configuration (e.g., GR24^5DS^). All three‐dimensional structure illustrations were generated using PyMOL Molecular Graphics System, Schrödinger, LLC. Displayed cavity volumes were measured using PyVOL (Smith et al., 2025) with default settings.

In animals, the mechanism of olfaction is well understood and mediated by membrane‐bound G‐protein‐coupled receptors (GPCRs) (Billesbølle et al., 2023; Jiang & Matsunami, 2015; Wicher & Miazzi, 2021). However, all studied putative GPCRs in plants appear not to be involved in VOC perception, with other compound‐specific receptor types implicated instead (Chakraborty & Raghuram, 2022; Giordano et al., 2021; Loreto & D'Auria, 2022; Urano & Jones, 2014). Indeed, a number of receptors and receptor‐like proteins for volatile compounds have been described including those for methyl salicylate (MeSA), methyl jasmonate (MeJA), ethylene, sesquiterpenes, and karrikins (KARs) (Figure 1; Table 1). A high‐affinity receptor for MeSA is the odorant‐binding protein (OBP)‐like receptor SALICYLIC ACID BINDING PROTEIN 2 (SABP2), which is a catalytic enzyme with esterase activity (Forouhar et al., 2005; Gong et al., 2023). It is a cytosolically localized member of the α/β hydrolase superfamily that demethylates MeSA to the phytohormone salicylic acid (SA) (Forouhar et al., 2005; Gong et al., 2023; Kumar & Klessig, 2003; Park et al., 2007; Soares et al., 2022; Vlot et al., 2008; Yao et al., 2015; Zhao et al., 2009). The Ser‐His‐Asp catalytic triad within SABP2 facilitates this hydrolysis inside the hydrophobic active site pocket that undergoes an open to closed transition, favoring substrate binding and product release (de Lima Silva et al., 2019; Forouhar et al., 2005) (Figure 2a). SABP2 has a particularly high binding affinity for SA, which leads to strong feedback inhibition of its activity by its own product (Park et al., 2009). This feedback inhibition can be modulated by a single polymorphic residue, Ala13 in *Nicotiana tabacum* (NtSABP2) and the corresponding Val18 in the *Citrus sinensis* SABP2 homolog protein (CsMES1), that also influences SABP2 catalytic efficiency (de Lima Silva et al., 2019; Forouhar et al., 2005). Indeed, site‐directed mutagenesis showed that residues with longer non‐polar side chains (e.g., Val) at this position decreased SA‐binding and feedback inhibition, whereas shorter (e.g., Ala) and/or polar side chains (e.g., Ser) led to a higher binding affinity for SA and correspondingly lower *V*_max_ and catalytic efficiency (de Lima Silva et al., 2019; Forouhar et al., 2005; Park et al., 2007). Interestingly, the SABP2 hydrophobic binding pocket contains several occluding aromatic amino acid residues that are a common feature of putative OBPs (Forouhar et al., 2005; Giordano et al., 2021). Moreover, *in silico* molecular docking simulations predicted that SABP2 may also bind volatile terpenoid compounds, although these receptor–ligand interactions have not been experimentally validated (Giordano et al., 2021). Upon binding of SABP2 with intracellular MeSA, the released free SA can then enter the nucleus and interact with multiple NONEXPRESSOR OF PATHOGENESIS‐RELATED GENES (NPR) receptors that have different SA‐binding activities (Fu et al., 2012; Wu et al., 2012; Yan & Dong, 2014). To date, the relative and specific contributions of the different NPRs to SA signaling remains incompletely resolved and the current status of the field is described in recent reviews (Peng et al., 2021; Spoel & Dong, 2024). In brief, SA binding with NPR1 and simultaneous suppression of NPR3/4 enables TGACG motif‐binding (TGA) and NAC2 (NAC refers to NAM, ATAF1/2, and CUC2) transcription factor‐mediated transcription of SA‐responsive genes, thus triggering systemic acquired resistance (Ding et al., 2018; Gong et al., 2023; Liu et al., 2020; Wang, Wang, et al., 2020). While SABP2 remains the best candidate receptor for airborne MeSA (Gong et al., 2023), SABP3, a plastidial β‐carbonic anhydrase (Medina‐Puche et al., 2017), and nearly 100 other proteins, many of which were identified through screening with SA‐analogs (Liao et al., 2015; Manohar et al., 2015; Moreau et al., 2013; Tian et al., 2012), may participate in MeSA perception and SA signaling (Pokotylo et al., 2019; Spoel & Dong, 2024). However, their physiological roles in SA signaling *in planta* remain to be fully elucidated.

**Table 1:** Proteins mediating volatile organic compound (VOC) perception in plants and associated ligand properties

Protein	Protein family	Ligand	Evidence for ligand perception	Ligand vapor pressure [Pa]^u^	Ligand Henry's law volatility constant [mol/(m^3^Pa)]
AtETR1	Ethylene receptors	Ethylene	Biochemical^a^ *In‐planta* ^a^	6.9 × 10^6^ ^b^	5.9 × 10^−5^ ^c^, ^v^
NtSABP2	α/β Hydrolase (methylesterase)	Methyl salicylate	Biochemical^d^ *In‐planta* ^e^	11^f^	1.6^g^, ^v^
NaMJE/VvMJE1	α/β Hydrolase (methylesterase)	Methyl jasmonate	Biochemical^h^ *In‐planta* ^i^	1.2 × 10^−1^ ^j^	50^k^, ^v^
AtMES2	α/β Hydrolase (methylesterase)	Methyl nicotinate	Biochemical^l^ *In‐planta* ^l^	18^m^	220^n^
NtTPL3	TOPLESS/TOPLESS RELATED	β‐Caryophyllene	Biochemical^o^ *In‐planta* ^o^	2.6^p^	3.7 × 10^−4^ ^q^, ^v^
PhKAI2ia	α/β Hydrolase	(−)‐Germacrene D	Biochemical^r^ *In‐planta* ^r^	1.3^s^	8.9 × 10^−4^ ^t^

^a^ Azhar et al. (2023).

^b^ Douslin and Harrison (1976).

^c^ Burkholder et al. (2019).

^d^ Forouhar et al. (2005).

^e^ Gong et al. (2023).

^f^ Yaws and Satyro (2015).

^g^ Brockbank (2013).

^h^ Zhao et al. (2016).

^i^ Wu et al. (2008).

^j^ Naef and Acree Jr. (2021).

^k^ Karl et al. (2008).

^l^ Wu et al. (2018).

^m^ da Ribeiro Silva et al. (2007).

^n^ Neely and Blau (1985).

^o^ Nagashima et al. (2019).

^p^ Orf et al. (2021).

^q^ Copolovici and Niinemets (2015).

^r^ Stirling et al. (2024).

^s^ Umnahanant and Chickos (2024).

^t^ Estimated by HENRYWIN v3.20 in EPI Suite™ version 4.0 software—US EPA. 2026. Estimation Programs Interface Suite™ for Microsoft^®^ Windows, v 4.11. United States Environmental Protection Agency, Washington, DC, USA.

^u^ At 298.15 K.

^v^ Preferred value and citation from Sander (2023).

Similar to SABP2 with MeSA, MeJA esterase (MJE) demethylates volatile MeJA in the cell to form JA (Koo et al., 2013; Stuhlfelder et al., 2002, 2004; Zhao et al., 2016). As a member of the α/β hydrolase superfamily, MJE is structurally homologous to SABP2 (Figure 2b) and contains the same conserved Ser‐His‐Asp catalytic triad but exhibits substrate promiscuity, accepting, in addition to MeJA, other methyl esters including MeSA, though with a lower efficiency (Han et al., 2006; Stuhlfelder et al., 2002; Zhao et al., 2016). In contrast to SABP2, which possesses bulky hydrophobic residues (Trp131, Met149, and Phe155) at the binding site and near the surface of the protein that render MeJA binding unfavorable, MJE possesses smaller residues at the corresponding positions (Ser131, Ile149, and Asp155), enabling accommodation of the larger substrate (Zhao et al., 2016). Jasmonic acid (JA) released in the cytosol is then conjugated to isoleucine by JASMONATE RESISTANT 1 (JAR1) and its homologs to form (+)‐7‐iso‐jasmonoyl‐L‐isoleucine (JA‐Ile) (Browse, 2009; Fonseca et al., 2009; Kang et al., 2006; Staswick & Tiryaki, 2004; Wang et al., 2007). JA‐Ile is imported into the nucleus by the JA/JA‐Ile transporter JASMONATE TRANSPORTER 1 (JAT1; AtABCG16 in Arabidopsis), a member of the ATP‐binding cassette G (ABCG) family of proteins that localizes to both the plasma membrane and nuclear envelope (Li et al., 2017; Wang et al., 2019). Once inside the nucleus, JA‐Ile binds to the F‐box protein CORONATINE INSENSITIVE1 (COI1), a part of a larger co‐receptor complex containing the transcriptional co‐repressor JASMONATE‐ZIM‐DOMAIN (JAZ) proteins (Chini et al., 2007; Howe et al., 2018; Sheard et al., 2010; Thines et al., 2007). The subsequent JAZ degradation releases repression of JA‐responsive genes, thereby enabling their transcriptional activation. However, MeJA perception and downstream signaling in the cell are impacted by other JA sources. JA is also produced in the peroxisome from 12‐oxophytodienoic acid (OPDA) or 4,5‐didehydrojasmonate (Acosta & Farmer, 2009; Chini et al., 2018) and exported to the cytosol where it joins the MeJA‐derived pool. Regardless of its source, cytosolic JA has several fates as it can be exported from the cell by JAT1, methylated by S‐adenosyl‐L‐methionine:jasmonic acid carboxyl methyltransferase (JMT) to MeJA (Seo et al., 2001; Yue et al., 2024; Zhao, Yao, et al., 2013), converted to JA‐Ile by JAR1, or conjugated with other carrier molecules. Moreover, in addition to being imported to the nucleus, JA‐Ile can be converted back into JA by jasmonoyl‐L‐isoleucine hydrolase 1 (JIH1) (Woldemariam et al., 2012, 2014) or catabolized/inactivated into 12‐OH‐JA‐Ile and 12‐COOH‐JA‐Ile by several members of the cytochrome P450 monooxygenase (CYP) 94 family (Heitz et al., 2012; Kitaoka et al., 2011; Koo et al., 2011, 2014). Multiple potential JATs further modulate the cytosolic‐nuclear partitioning of JA and JA‐Ile to regulate JA‐dependent responses (Wang et al., 2019). While the canonical signaling pathway is generally conserved among land plants, the ligand specificity of COI1‐JAZ co‐receptor complexes varies due to polymorphisms in both COI1 and JAZ (Bowman et al., 2017; Monte et al., 2018, 2022). Angiosperm COI1‐JAZ complexes utilize JA‐Ile while those in liverwort (*Marchantia polymorpha*) use dn‐cis‐OPDA, dn‐iso‐OPDA, and Δ4‐dn‐iso‐OPDA instead (Chini et al., 2023; Kneeshaw et al., 2022; Monte et al., 2018, 2022). Furthermore, lycophytes and bryophytes likely use dn‐iso‐OPDA as the ligand for COI1‐JAZ co‐receptors, suggesting that it is an ancestral ligand that predates the employment of JA‐Ile in angiosperms (Chini et al., 2023; Pratiwi et al., 2017).

In addition to SABP2 and MJE, several other methylesterases in plants may serve similar functions in initiating downstream responses to volatile and semi‐volatile signals, including Arabidopsis AtMES2 for methyl nicotinate, a volatile long‐distance transport form of the nicotinamide adenine dinucleotide precursor nicotinate (Wu et al., 2018), as well as numerous MeJA/MeSA esterases among other activities (Chaffin et al., 2024). Although they are not conventional membrane‐bound receptors, these methylesterases act as functional receptor‐like enzymes wherein the perception of the volatile compounds occurs through the enzymatic conversion of the volatile into a subsequent, non‐volatile active signaling molecule. This methylesterase‐receptor activity occurs within a land‐plant‐specific group of the α/β hydrolase superfamily often involved in phytohormone signaling pathways, offering a potentially rich source of receptor‐like proteins for VOC perception. Indeed, these methylesterases have diverse functions, and several groups remain largely unexplored (Chaffin et al., 2024).

The perception of volatiles is not limited to enzymatic conversion of VOCs by soluble methylesterases. Ethylene is perceived by a group of partially redundant Cu^2+^‐dependent ethylene receptors located in the membrane of the endoplasmic reticulum (ER) (Chang et al., 1993; Chen et al., 2002; Rodríguez et al., 1999; Schaller & Bleecker, 1995). The details of ethylene perception and signal transduction have been thoroughly covered in prior reviews (Azhar et al., 2020; Binder, 2020; Zhao et al., 2020). The ethylene receptors, which typically act as dimers, contain an N‐terminal ligand‐binding domain embedded in the ER membrane, as well as cytosolically located histidine kinase‐like and cGMP phosphodiesterase/adenylyl cyclase/FhlA (GAF) domains, and sometimes an additional regulatory receiver domain (Azhar et al., 2023; Gallie, 2015; Ju & Chang, 2015). Ethylene receptors are divided into two subfamilies with mostly overlapping functions, but with distinct impacts on the larger receptor complex stability (Berleth et al., 2019). In general, subfamily I members (e.g., ETHYLENE RESPONSE 1; ETR1, ETHYLENE RESPONSE SENSOR 1; ERS1) have an intact histidine kinase domain, whereas those of subfamily II (e.g., ETR2, ERS2, ETHYLENE‐INSENSITIVE 4; EIN4, *N. tabacum* HISTIDINE KINASE 1; NTHK1) have a serine/threonine kinase domain and include the additional receiver domain (Gamble et al., 1998; Moussatche & Klee, 2004; Wang et al., 2006; Xie et al., 2003). Upon ethylene binding, the receptor protein complex undergoes a conformational change that deactivates the Raf‐like serine/threonine protein kinase CONSTITUTIVE TRIPLE RESPONSE1 (CTR1). This CTR1 inactivation allows dephosphorylation of EIN2, which is then proteolytically cleaved, releasing its C‐terminal domain (EIN2‐CEND) that is translocated to the nucleus to activate downstream transcriptional responses (Berleth et al., 2019; Binder, 2020; Clark et al., 1998; Huang et al., 2003; Ju et al., 2012; Qiao et al., 2009; Wang et al., 2006; Wen et al., 2012). In the nucleus, EIN2‐CEND facilitates EIN3 and EIN3‐LIKE (EIL)‐induced transcriptional activation by interacting with EIN2 NUCLEAR‐ASSOCIATED PROTEIN 1 (ENAP1) that mediates histone acetylation of target DNA regions (Zhang et al., 2016, 2017). EIN2‐CEND also indirectly stabilizes the transcription factor EIN3/EIL and prevents their targeting by EIN3‐binding F‐box proteins (EBFs) for degradation by repressing EBF translation (Li et al., 2015; Merchante et al., 2015), thus enabling EIN3/EIL‐mediated transcriptional activation of ethylene‐responsive genes including other transcription factors such as *ETHYLENE RESPONSE FACTOR*s (ERFs) (Chang et al., 2013). As part of a secondary ethylene response, EIN3 can be stabilized by CTR1 that was recently shown to translocate to the nucleus in response to ethylene, where it interacts with and inhibits EBF proteins (Park, Nam, & Park, 2023). In the absence of ethylene, EIN2 is phosphorylated by CTR1 into a less stable inactive state that is targeted by F‐box proteins (EIN2 TARGETING PROTEIN 1 [ETP1] and ETP2) for 26S proteasomal degradation (Binder, 2020; Ju et al., 2012; Qiao et al., 2009; Wen et al., 2012). Although ethylene signaling may also proceed through CTR1‐independent pathways, signaling through the CTR1‐dependent pathway is partially regulated by the phosphorylation state of CTR1 and by the levels of sterols in the ER membrane (reviewed in Zhao et al. (2020)). Furthermore, ethylene receptor availability can be regulated by a recently discovered ethylene‐dependent ER‐associated degradation mechanism (ERAD) where subfamily II ethylene receptors are targeted for degradation by an E3 ligase RING finger for ethylene receptor degradation (RED) and a conserved E2 conjugating enzyme UBIQUITIN‐CONJUGATING ENZYME 32 (Zhao et al., 2025).

While plant perception of volatile hormones/growth regulators (e.g., MeJA, MeSA, ethylene) has been extensively studied and the downstream signaling events are well understood, the receptors for volatile secondary metabolites, including terpenoids, remain much less explored. Thus far, the two known terpenoid sensing mechanisms do not appear to require membrane‐bound receptors or soluble methylesterases and instead rely on functionally distinct proteins. TOPLESS‐like proteins (TPLs), a group of transcriptional co‐repressors, were found to bind to caryophyllene derivatives using pulldown assays (Nagashima et al., 2019), though interacting partners and regulatory mechanisms involved remain unknown. Overexpression of *NtTPL3* fused with the coding sequence of green fluorescence protein dampened the transcriptional response of tobacco plants and BY‐2 cells to caryophyllene‐oxide; however, the role of TPLs in perception of other terpenoids is yet to be determined, and the presence of TPL VOC receptors in other plants has not yet been shown. Recently, the hormone‐like function of terpenoids was shown in petunia flowers where the volatile sesquiterpene (−)‐germacrene D, produced by a tube‐specific terpene synthase (PhTPS1), was perceived in developing pistils, and required for normal pistil development and seed yield (Boachon et al., 2019; Stirling et al., 2024). The perception of (−)‐germacrene D was found to be stereospecifically mediated by a clade‐specific KARRIKIN INSENSITIVE 2 (KAI2) receptor (Stirling et al., 2024) through a KAR‐like signaling pathway that partially overlaps with but is distinct from the canonical KAR signaling pathway.

The KAI2 receptors represent one of the only other known groups of VOC receptors in plants. They exhibit dual localization in the cytosol and nucleus (Khosla, Morffy, et al., 2020; Stirling et al., 2024) and are members of the α/β hydrolase superfamily but are distinct from the methylesterase group that includes SABP2 and MJE. They were initially found through their perception of KARs, a class of butenolide compounds released from wildfire smoke that play a role in post‐fire seed germination (Flematti et al., 2004, 2015; Nelson et al., 2012). Since their initial discovery and despite not being produced by plant metabolism, KARs have been shown to elicit a wide range of responses across the plant kingdom through their perception by KAI2 receptors (Bonhomme & Guillory, 2022; Park, Seo, et al., 2023; Varshney & Gutjahr, 2023; Waters & Nelson, 2023). However, as KAI2s are conserved across land plants and KAI2‐like proteins are even found in charophyte algae, the primary function of KAI2s is likely to sense an alternate, endogenous ligand (termed KAI2 ligand(s) or KL) of unknown origin (Bythell‐Douglas et al., 2017; Delaux et al., 2012; Walker et al., 2019; Waters et al., 2012). The growing interest in KAI2‐mediated signaling and the discovery of an intermediate clade KAI2 that senses a volatile sesquiterpene warrant further dissection of the mechanisms underlying KAI2‐mediated volatile ligand perception (Conn & Nelson, 2016; Stirling et al., 2024). Therefore, the remainder of this review will focus on recent discoveries and structural insights surrounding KAI2 receptors and their perception of currently known ligands.

## STRUCTURAL AND FUNCTIONAL DIVERSITY OF KAI2 RECEPTORS

KAR/KL signaling is initiated by the KAI2/HYPOSENSITIVE TO LIGHT (HTL) receptors that are found throughout the plant kingdom (recently reviewed in Waters and Nelson (2023)). Phylogenetic analysis of KAI2s revealed the presence of at least three clades that emerged in the lamiids (~50 000 species; Yang et al., 2020), each following distinct evolutionary trajectories within the larger D14/KAI2 family that also includes strigolactone (SL) receptors (e.g., DWARF14 (D14)/DECREASED APICAL DOMINANCE2 (DAD2)/RAMOSUS3 [RMS3]) (Bythell‐Douglas et al., 2017; Conn et al., 2015). These clades have unique structural features and ligand specificities and are designated as conserved (KAI2c), intermediate (KAI2i), and divergent (KAI2d), with members of the KAI2c clade subjected to the strongest purifying selection among them. The general structural features and ligand interactions of KAI2c receptors have been analyzed in detail, often using Arabidopsis AtKAI2 as the archetype (Figure 2d) (Bythell‐Douglas et al., 2013; Conn & Nelson, 2016; Guercio et al., 2022, 2024; Guo et al., 2013; Kushihara et al., 2025; Lee et al., 2018; Zhao, Zhou, et al., 2013). The AtKAI2 crystal structure shows a high degree of similarity to D14 with an α/β hydrolase fold containing a seven‐stranded β‐sheet (β2–β8) surrounded by helices (Bythell‐Douglas et al., 2013; Zhao, Zhou, et al., 2013). The helical lid domain between β6 and β7 strands creates a hydrophobic binding pocket with a catalytic Ser95‐His246‐Asp217 triad where KARs, as ligands, interact with Arabidopsis KAI2 through π–π stacking interactions of its butenolide ring with nearby aromatic residues (Guo et al., 2013). KAI2c receptors maintain a conserved ligand binding pocket size and shape, partly due to a series of conserved residues, including Tyr124, that restrict its internal volume (Figure 2d) (Conn et al., 2015; Guercio et al., 2024; Martinez et al., 2022).

Unlike the conserved clade, the KAI2i clade has undergone specialization that resulted in structural changes and altered ligand specificity (Conn & Nelson, 2016; Martinez et al., 2022; Xu et al., 2016). While most lamiids containing the intermediate clade possess only a single copy of KAI2i (Conn & Nelson, 2016), members of the Solanaceae typically harbor two genes in each of the conserved and the intermediate KAI2 clades (Stirling et al., 2024; Xu et al., 2022). Computational and homology‐based modeling predicted that KAI2i proteins exhibit intermediate structural morphology between KAI2c and D14, with the KAI2i ligand‐binding cavity typically being larger than that of KAI2c receptors (Conn et al., 2015; Takei et al., 2023). The Tyr124 residue of the KAI2c clade (numbering based on AtKAI2) is substituted with Phe124 in most KAI2i proteins as in D14, thus preventing Tyr124‐mediated restriction of the pocket adjacent to the active site and expanding the cavity volume (up to 189 Å^3^ in intermediate clade versus 126 Å^3^ in AtKAI2) (Conn et al., 2015; Martinez et al., 2022). Moreover, three critical pocket residues in addition to Tyr124 in AtKAI2 (Val96, Leu139, Ala161) that influence the binding pocket size are substituted by a typical KAI2i‐specific consensus combination (Leu96, Phe124, Ile139, Val161) that also includes a conserved Phe190 (Figure 2d) (Martinez et al., 2022). These structural differences, in addition to those listed in Table 2, likely alter the ligand specificity of the KAI2i clade, expanding its repertoire of signaling molecules. Examples include ShKAI2i proteins from *Striga hermonthica*, which bind KAR_1_ but not SLs (Conn et al., 2015; Conn & Nelson, 2016; Xu et al., 2016, 2018), as well as petunia PhKAI2ia that stereospecifically perceives a novel ligand, the olefinic sesquiterpene (−)‐germacrene D (Stirling et al., 2024).

**Table 2:** Key residues affecting the ligand specificity and hydrolysis of KAI2 receptor

Position (AtKAI2)	Typical residues in KAI2	Variation among KAI2 proteins	Functional role and significance	References
25	Phe25/Gly25	Phe25 is an ‘intrusive’ residue that limits the volume of the conserved KAI2 pocket, whereas Gly25 in PhKAI2ia	Forms the pocket entrance Gly25 coordinates the interaction of (−)‐germacrene D within the PhKAI2ia active site	Stirling et al. (2024)
26	Tyr26/Phe26	Phe26 in PhKAI2ia Tyr26 is one of eight ShHTL7 residues substituted into KAI2 to identify SL binding	Defines the pocket, specifically coordinating the D‐OH ring product in PsKAI2B Phe26 coordinates (−)‐germacrene D in PhKAI2ia	Arellano‐Saab et al. (2021) Stirling et al. (2024)
95	Ser95 (catalytic triad)	Ser95	Part of the conserved Ser‐His‐Asp catalytic traid. Essential for hydrolytic activity and KAR/KL responses Forms a transient covalent adduct with the D‐OH ring product upon ligand cleavage in PsKAI2B	Guercio et al. (2022)
96	Val96	Leu96 or Met96 in some specialized KAI2 proteins	Val96Leu substitution alone can reduce KAR_2_ responses Leu96 coordinates (−)‐germacrene D in PhKAI2ia Val96 in PsKAI2B coordinates the D‐OH product	Martinez et al. (2022) Stirling et al. (2024) Guercio et al. (2022)
124	Tyr124	Phe124 in Phe124‐type asterid KAI2 (e.g., LsKAI2b, ShKAI2i) Phe124 in PhKAI2ia Val124 in *Striga hermonthica* KAI2d ShHTL5	Key determinant of specificity Tyr124Phe substitution is often sufficient to reduce or abolish KAR_2_ response Phe124 is distinctly conserved within KAI2i clade proteins Val124 in ShHTL5 deepens the active site cleft Phe124 coordinates (−)‐germacrene D in PhKAI2ia	Martinez et al. (2022) Stirling et al. (2024) Toh et al. (2015)
134	Phe134	Phe134 in PhKAI2ia	Phe134 coordinates (−)‐germacrene D in PhKAI2ia Located near the entrance, it contributes to clogging the hydrophobic cavity in ShKAI2iB	Stirling et al. (2024) Xu et al. (2016)
139	Leu139	Conserved as aliphatic hydrophobic residues (e.g., Ile139 or Val139) Val139 in LsKAI2b and ShKAI2i	Substitution Leu139Ile may abolish KAR_2_ perception. Key position distinguishing KAI2 clades in asterids	Martinez et al. (2022)
142	Leu142	Conserved as aliphatic hydrophobic residues (e.g., Val142, Ile142) Leu142 in PhKAI2ia	In ShKAI2iB, Leu142 stabilizes the inward movement of the gatekeeper helix L142 coordinates (−)‐germacrene D in PhKAI2ia	Xu et al. (2016) Stirling et al. (2024)
153	Trp153 in KAI2 Met153 in D14/KAI2d	Trp153Leu substitution in gain‐of‐function SL receptor Var64	Substitution Trp153Leu is one of three required mutations to convert KAI2 into an SL receptor Met153 in ShHTL5 is involved in positioning the KAR in AtKAI2	Arellano‐Saab et al. (2021) Toh et al. (2015)
157	Phe157	Phe157Thr substitution in gain‐of‐function SL receptor Var64	A hotspot for diversification in the pocket region Phe157 is located near the entrance, contributing to clogging the hydrophobic cavity in ShKAI2iB Phe157Thr substitution in ShHTL7 and in Var64 correlates with KAI2 changes into SL receptor Phe157 coordinates (−)‐germacrene D in PhKAI2ia	Martinez et al. (2022) Xu et al. (2016) Arellano‐Saab et al. (2021) Stirling et al. (2024)
161	Ala161/Cys161	Val161 in Phe124‐type asterid KAI2 Val161 in PhKAI2ia Leu160 in PsKAI2A and Met160 in PsKAI2B are critical diverged residues	Ala161Val substitution is one of four residues noted to influence ligand specificity in asterids Val161 coordinates (−)‐germacrene D in PhKAI2ia	Martinez et al. (2022) Stirling et al. (2024) Guercio et al. (2022)
190	Gly190/Ala190	Gly190Thr substitution in gain‐of‐function SL receptor Var64 Leu190 in PsKAI2B Phe190 in Phe124‐type asterid KAI2	This position affects structural arrangement and pocket volume/hydrophobicity Thr190 in Var64 may play a stabilizing role, permitting other substitutions during evolution A hotspot for diversification	Guercio et al. (2022) Arellano‐Saab et al. (2021) Martinez et al. (2022)
194	Phe194/Cys194	Ser194 in ShHTL5 Phe194 in PhKAI2ia	Phe194 coordinates (−)‐germacrene D in PhKAI2ia	Stirling et al. (2024)
217	Asp217	Asp217	Part of the conserved Ser‐His‐Asp catalytic traid. Essential for hydrolytic activity and KAR/KL responses	
218	Leu218	Conserved with hydrophobic residues (e.g., Leu, Met, Ile) Leu218 in PsKAI2B and PhKAI2ia Met218 in PsKAI2A	Located in the D‐loop, suggested to be involved in downstream protein–protein interactions (e.g., with MAX2/SMXL2) L218 coordinates (−)‐germacrene D in PhKAI2ia	Guercio et al. (2022) Stirling et al. (2024)
246	His246	His246	Part of the conserved Ser‐His‐Asp catalytic traid Forms the stable covalent adduct with the D‐ring derived cleavage product (CLIM) of SL and potentially KL Coordinates (−)‐germacrene D in PhKAI2ia	Kushihara et al. (2025) de Saint Germain et al. (2016) Stirling et al. (2024)

Another phylogenetic branch of KAI2s, the KAI2d clade represents a highly specialized lineage that arose specifically within obligate parasitic plants of the Orobanchaceae family (Conn et al., 2015; Conn & Nelson, 2016; Takei et al., 2023; Yoshida et al., 2019). Within this plant family, most KAI2s belong to the divergent clade and have multiple copies with an overall greater number of KAI2 paralogs than is typical for non‐parasitic plants (Conn et al., 2015; Yoshida et al., 2019). KAI2d receptors predominantly detect SLs exuded from host plants, which triggers a strong germination response (Conn et al., 2015; Fernández‐Aparicio et al., 2011; Nelson, 2021; Rahimi & Bouwmeester, 2021; Toh et al., 2015; Tsuchiya et al., 2015; Wang et al., 2022). Homology models predict that KAI2d ligand‐binding pockets are larger than those of both KAI2c and KAI2i, partially due to substitutions of Tyr124 by small hydrophobic amino acids (e.g., Ile, Leu, Val, Met) (Figure 2d) (Arellano‐Saab et al., 2021; Conn et al., 2015; Martinez et al., 2022; Takei et al., 2023).

## CANONICAL KAR SIGNALING

In the KAI2‐mediated signaling pathway, that shares many common features with SL signaling, KAR molecules (or its derivatives) or the endogenous KL first bind to the KAI2 receptor (Guo et al., 2013; Morffy et al., 2016; Nakamura et al., 2013; Nelson et al., 2011; Sun & Ni, 2011; Waters et al., 2012; Waters, Scaffidi, Flematti, & Smith, 2015; Xu et al., 2016, 2018; Yao et al., 2018). Both KAI2 and D14 possess dual hydrolase/receptor activities and share the conserved Ser‐His‐Asp/Glu catalytic triad (also observed in SABP2 and MJE; Figure 2a–d), yet they display distinct ligand preferences (Figure 2d) (de Saint Germain et al., 2016; Hamiaux et al., 2012; Scaffidi et al., 2012; Tsuchiya et al., 2015; Uraguchi et al., 2018; Waters & Nelson, 2023; Waters, Scaffidi, Flematti, & Smith, 2015; Yao et al., 2018, 2021; Zhao, Zhou, et al., 2013). While KAI2c specifically interacts with the 2′S configuration of synthetic SLs (e.g., GR24^ent‐5DS^ or (−)‐GR24), D14 and KAI2d proteins are sensitive to 2′R‐configured SLs (e.g., GR24^5DS^ or (+)‐GR24) (Arellano‐Saab et al., 2021; Conn et al., 2015; Flematti et al., 2016; Guercio et al., 2022; Nelson, 2021; Scaffidi et al., 2014; Toh et al., 2015; Tsuchiya et al., 2015; Wang et al., 2022). Upon binding to the receptor, the ligand undergoes enzymatic hydrolysis which was confirmed by structural studies of *Pisum sativum* PsKAI2B with GR24^ent‐5DS^, and the detection of a transient catalytic intermediate attached to Ser95 and a covalent ligand adduct on His246 (Guercio et al., 2022). Similarly, KAI2‐like proteins from the moss *Physcomitrium patens* exposed to GR24^ent‐5DS^ were found with covalent ligand adducts on the corresponding histidine of the catalytic triad (Bürger et al., 2019; Lopez‐Obando et al., 2021). Ligand binding and hydrolysis is suggested to trigger conformational changes of both D14 and KAI2 receptors, leading to their subsequent interaction with the F‐box protein MORE AXILLARY GROWTH2 (MAX2), a component of the Skp, Cullin, F‐box (SCF) E3 ubiquitin ligase complex (Shabek et al., 2018; Stirnberg et al., 2007; Tal et al., 2020, 2023; Yao et al., 2016). This facilitates ubiquitination and 26S proteasomal degradation of transcriptional co‐repressors, SUPPRESSOR OF MAX2 1‐LIKE (SMAX1/SMXL2 in KL pathway and SMXL6/7/8 in SL pathway) proteins, leading to downstream physiological responses (Jiang et al., 2013; Stanga et al., 2013, 2016; Zhou et al., 2013).

## ENDOGENOUS KAI2 LIGANDS

**Figure 3:**
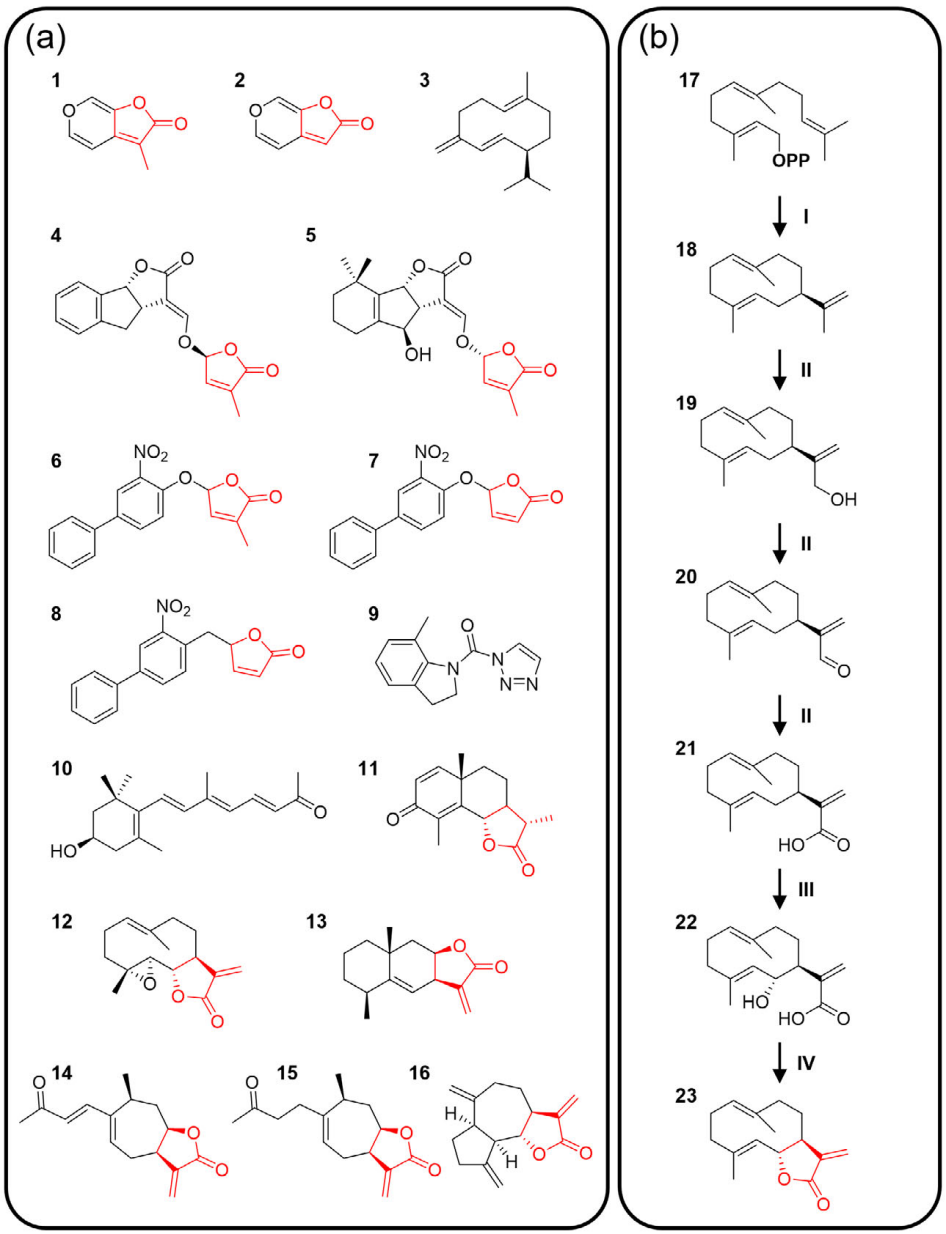
Chemical diversity of KAI2‐interacting compounds. (a) Molecules shown to bind to KAI2 proteins. **1**, KAR_1_ and **2**, its desmethyl version KAR_2_. **3**, (−)‐germacrene D. **4**, GR24^ent‐5DS^/(−)‐GR24 and **5**, orobanchol, strigolactones. **6**, germinone A and its desmethyl **7**, dMGer and non‐hydrolyzable **8**, (+)‐6'‐carba‐dMGer derivatives. **9**, KK181N1, a known KAI2 antagonist. **10**, α‐zaxinone, an apocarotenoid. **11**, (−)‐α‐santonin; **12**, parthenolide; **13**, alantolactone and **23**, costunolide, sesquiterpene lactones. **14**, 8‐*epi*‐xanthatin and **15**, tomentosin, two sesquiterpene lactones shown *in silico* to bind to KAI2. **16**, (−)‐dehydrocostus lactone, a sesquiterpene lactone shown to covalently bind to KAI2. (b) Scheme showing the known steps for costunolide biosynthesis as a representative sesquiterpene lactone (reviewed in Frey et al. (2024)). **17**, (*E*, *E*)‐farnesyl diphosphate; **18**, (+)‐germacrene A; **19**, (+)‐germacrene A alcohol; **20**, (+)‐germacrene A aldehyde; **21**, (+)‐germacrene A acid; **22**, (+)‐6α‐hydroxy germacrene A acid; **23**, (+)‐costunolide; **I**, germacrene A synthase; **II**, germacrene A oxidase (CYP71AV); **III**, costunolide synthase (CYP71BL); **IV**, spontaneous. **OPP**, diphosphate.

Although KARs (Figure 3a #**1**, **2**) are abiotic in origin (Flematti et al., 2004), they are bioactive and able to interact with KAI2 *in vitro* (Bürger & Chory, 2020; Guercio et al., 2022; Guo et al., 2013; Kagiyama et al., 2013; Lee et al., 2018; Xu et al., 2016), suggesting that KARs, and possibly their downstream metabolic products, mimic the yet unidentified authentic KL (Conn & Nelson, 2016; Khosla, Rodriguez‐Furlan, et al., 2020; Sepulveda et al., 2022; Wang, Withers, et al., 2020; Waters, Scaffidi, Flematti, & Smith, 2015; Xu et al., 2018). The known interactions of KARs and synthetic SL analogs (e.g., GR24^ent‐5DS^; Figure 3a #**4**) with KAI2 and the similarity of KAI2 to D14 have enabled the elucidation of potential structural requirements for KL, which is postulated to include a hydrolyzable butenolide moiety that is processed by the catalytic triad of KAI2 (Arellano‐Saab et al., 2021; de Saint Germain et al., 2016; Guercio et al., 2022; Kushihara et al., 2025; Martinez et al., 2022; Yang et al., 2019; Yao et al., 2016). Moreover, KAI2s from both vascular (lycophyte; *Selaginella moellendorffii*) and non‐vascular (liverwort; *M. polymorpha*) plants were shown to be more sensitive to KARs with butenolide rings lacking the 4′‐methyl group (Figure 3a #**2**; Yao et al., 2021; Meng et al., 2022), although the methyl group on the butenolide ring of SLs is essential for interaction of the ligand with D14 (Figure 3a #**4**, **5**; Boyer et al., 2012; de Saint Germain et al., 2016). The ligand potencies were further analyzed by testing additional synthetic compounds that selectively bind to KAI2, including germinone A (synthetic SL analog, Ger; Figure 3a #**6**) and its desmethyl (dMGer; Figure 3a #**7**) and non‐hydrolyzable (carba‐dMGer; Figure 3a #**8**) derivatives (Fukui et al., 2019; Kushihara et al., 2025; Okabe et al., 2023), as well as the KAI2 antagonist KK181N1 (Figure 3a #**9**), a triazole urea derivative that lacks a butenolide moiety (Wang et al., 2025). Indeed, germinone A, which has a methylated butenolide moiety appeared to be less potent and less specific than its desmethyl derivative (Fukui et al., 2019; Okabe et al., 2023), and the non‐hydrolyzable carba‐dMGer could not induce KAI2‐mediated signaling (Kushihara et al., 2025). Furthermore, KK181N1 which cannot be hydrolyzed by KAI2, competes for the KAR binding site and antagonistically inhibits KAI2 (Wang et al., 2025). Additionally, zaxinone (Figure 3a #**10**), an endogenously produced apocarotenoid derived from β‐carotene and lacking a butenolide moiety, was recently shown to interact with D14, and to a lesser extent, KAI2 receptors as a non‐hydrolyzable antagonist and repress KAI2‐dependent gene expression (Moreno et al., 2025). However, PrKAI2d3 from the parasitic plant *Phelipanche ramosa* shows high plasticity, allowing it to covalently bind isothiocyanates in addition to 2′R, but not 2′S configured strigolactones (de Saint Germain et al., 2021). Taken together, these results suggest that KAI2s generally prefer ligands with a hydrolyzable butenolide moiety lacking a 4′‐methyl group for specific activation and downstream signaling, often exhibiting stereoselectivity, but plasticity in KAI2 structures may allow varied ligand preferences in some cases.

Recently the olefinic sesquiterpene (−)‐germacrene D (Figure 3a #**3**) was identified as the first endogenous KL for a specialized KAI2 receptor variant (PhKAI2ia) of the intermediate clade in petunia (Stirling et al., 2024) (Figure 2). PhKAI2ia stereospecifically interacts with (−)‐germacrene D to activate a KAR‐like signaling cascade that involves MAX2 (PhMAX2a and/or PhMAX2b) and leads to proteolytic degradation of PhSMAX1a, ultimately inducing a transcriptional response that shares some, but not all of the common KAR‐responsive genes. (−)‐Germacrene D is produced in developing petunia petal tubes and *PhKAI2ia* expression in the pistils is sensitive to its level (Boachon et al., 2019; Stirling et al., 2024). In line with prior observations that KAI2 shows stereoselective ligand preferences (Arellano‐Saab et al., 2021; Conn & Nelson, 2016; Guercio et al., 2022; Scaffidi et al., 2014; Villaécija‐Aguilar et al., 2022; Waters, Scaffidi, Moulin, et al., 2015; Yao et al., 2021), only (−)‐germacrene D and not its (+)‐enantiomer was shown to interact with PhKAI2ia, and only (−)‐germacrene D out of 10 tested compounds including KARs, terpenoids, and a phenylpropanoid, was able to rescue the small pistil phenotype of *PhTPS1*‐RNAi plants (Boachon et al., 2019; Stirling et al., 2024). Remarkably, this is the only example to date demonstrating interaction of an olefinic ligand with any member of the KAI2 family of receptors. PhKAI2ia exhibits a divergent ligand specificity, acting as a specialized receptor for (−)‐germacrene D, to which the conserved clade member (PhKAI2ca) does not respond (Stirling et al., 2024). The stereoselectivity of PhKAI2ia for (−)‐germacrene D over (+)‐germacrene D likely reflects structural constraints within the binding pocket driven by its volume, overall shape, and the precise positions of critical amino acids relative to the ligand, similar to what has been documented for KAI2c and KAI2d with GR24^ent‐5DS^ versus GR24^5DS^ (Arellano‐Saab et al., 2021; Takei et al., 2024; Yao et al., 2021). Nevertheless, since the majority of experiments to ascertain the role of (−)‐germacrene D in PhKAI2ia‐mediated signaling were performed *in vivo*, it is possible that (−)‐germacrene D is further metabolized into a more potent ligand (e.g., germacrene lactone). Indeed, it has been demonstrated that sesquiterpene lactones (STLs) (e.g., α‐santonin; Figure 3a #**11**–**13**, **23**) can be perceived by KAI2d proteins in the parasitic plants *Striga* and *Orobanche*, and they mediate germination initiation (Joel et al., 2011; Raupp & Spring, 2013; Takei et al., 2023; Wu et al., 2022) and chemotropic growth (Krupp et al., 2021). Additionally, *in silico* molecular docking analysis with endogenous STLs from *Helianthus annuus* (sunflower) suggests that 8‐*epi*‐xanthatin and tomentosin (Figure 3a #**14**, **15**) but not dehydrocostus lactone (DCL) and costunolide (Figure 3a #**16**, **23**), may function as ligands for sunflower KAI2 receptor(s) *in planta* (Rahimi & Bouwmeester, 2021). In contrast, it has been recently shown that DCL can covalently bind to both *Orobanche cumana* OcKAI2d2 and AtKAI2 through two conserved catalytic serine residues, as demonstrated by LC–MS analysis of purified proteins with 500 μM DCL. Furthermore, DCL was shown to have a similar *K*_d_ (3.56 μM) for AtKAI2 as *rac*‐GR24 (2.67 μM) (Han et al., 2024). To date, the *in planta* impact and biological significance of DCL within the KAR‐signaling pathway remains unresolved.

Thus far, at least 158 volatile sesquiterpenes (114 of which are cyclic) are known to be produced by multiple plant families (Knudsen & Gershenzon, 2020). A subset of these sesquiterpenes can be further metabolized into highly bioactive STLs which function as protective compounds by deterring herbivores, acting as allelochemicals, alleviating ozone damage, and inhibiting growth of microbial pathogens (Chadwick et al., 2013; Frey et al., 2024; Macías et al., 1996; Rodriguez et al., 1976). They often accumulate to high levels in glandular trichomes, latex, or secretory ducts, particularly within the Asteraceae family, and are also found sporadically in many other plant families (e.g., Apiaceae, Winteraceae, Orchidaceae, and Lauraceae) (Chadwick et al., 2013; Frey et al., 2024; Padilla‐Gonzalez et al., 2016; Picman, 1986; Rodriguez et al., 1976). Beyond their protective roles, STLs are now emerging as endogenous signaling molecules that modulate growth and developmental processes (Frey et al., 2024), and their biosynthesis appears developmentally and spatially restricted (Spring et al., 2020). STLs are produced from C15 sesquiterpene skeletons such as germacranolides, guaianolides, eudesmanolides, pseudoguaianolides, and others (reviewed in Padilla‐Gonzalez et al. (2016) and Agatha et al. (2024)) through a series of oxidation steps starting with the addition of a primary hydroxyl group by a CYP (likely CYP71), formation of an aldehyde intermediate and then acid, followed by a final cyclization to form the characteristic lactone ring (Figure 3b) (Zhang, Wu, et al., 2023). The synthesized STLs can be further decorated by CYPs, dehydrogenases, and acyltransferases, expanding the range of their bioactivities and ecological functions. A defining structural feature of STLs is a reactive α‐methylene‐γ‐lactone group (butenolide ring with an exocyclic methylene) responsible for many of their bioactivities and making them attractive lead compounds for potential KLs. Therefore, the elucidation of factors determining KAI2 ligand specificity and the mechanistic basis for KAI2 activation, as well as detailed structural analyses of KAI2 signaling complexes in the presence and absence of ligands with different properties, are required.

## PERSPECTIVES AND FUTURE DIRECTION

To date, it has been extensively documented that plants perceive volatile compounds from the surrounding environment, yet a detailed mechanistic understanding of how they perceive these chemical cues and convert them into biological signals as well as corresponding physiological responses is still lacking. While plants produce, emit, and perceive numerous structurally diverse VOCs released from multiple biotic and abiotic sources, only a few receptors have been identified and characterized thus far. These receptors include membrane‐bound ethylene receptors, a transcriptional co‐repressor, and members of the α/β hydrolase superfamily, with no clear group or phylogenetic separation defining VOC receptors, illustrating a multifaceted nature and complexity of plant volatile sensing and communication. Furthermore, the small number of known and putative VOC receptors and OBPs cannot account for the vast number of volatile chemical signals known to be sensed by plants. From those that have been characterized, several plant VOC receptors appear to be functional enzymes with hydrolase/esterase activity yielding a less volatile, but more potent signal which initiates the downstream signaling cascade (Box 1). It remains unclear if plants, (i) similar to animals, use a large suite of conventional, but yet unidentified OBPs, (ii) detect VOCs through their enzymatic conversion into metabolic signals (e.g., MeSA to SA, MeJA to JA to JA‐Ile, sesquiterpenes to STLs), or (iii) a combination of both strategies. Nevertheless, given the cytosolic localization of most identified VOC receptors, an open question is how these lipophilic molecular signals encounter their target receptor(s) (Box 2). This might involve, in addition to the contribution of passive diffusion, facilitated transport by LTPs similar to emission (Liao et al., 2023), partitioning into vesicle bilayers, or shuttling by unknown carriers.

Box 1 **Main summary points**

- Plants perceive VOCs that play roles in defense, development, and communication.
- Identified plant VOC receptors include methylesterases (e.g., SABP2), ethylene receptors, transcriptional co‐repressors (e.g., TOPLESS‐like proteins), and α/β hydrolases of the KAI2 family.
- VOC perception often involves enzymatic conversion of volatile compounds into more potent signaling molecules that trigger downstream responses.
- Different clades of KAI2 receptors possess distinct structural features that define their specific ligand‐binding preferences.
- Emerging evidence links volatile terpenoid perception to KAI2‐mediated signaling, a previously unrecognized role for this pathway.

Box 2 **Main open questions**

- What molecular mechanisms determine the specificity of plant perception and responses to the diverse array of volatile signals?
- How are volatile compounds transported and delivered to cytosolically localized receptors?
- Are volatile compounds like (−)‐germacrene D perceived directly by KAI2 receptors or converted into lactones before receptor activation *in planta*?
- How do structural dynamics of KAI2‐MAX2‐SMAX1 complexes with different ligands determine the specificity of transcriptional outputs?
- What other types of receptors might plants use to sense the wide range of volatile compounds?

Current experimental evidence suggests that KAI2‐type α/β hydrolase receptors also perceive volatile compounds such as (−)‐germacrene D and KARs. However, it is possible that some volatiles do not directly activate KAI2s but are first metabolically converted into more potent ligands (e.g., lactones) that serve as the true signaling compounds. If this conversion is indeed a prerequisite for KAI2 activation, these derivatives could represent a key biochemical link between volatile perception and downstream signaling in plants. However, it remains unknown how many volatile signals are perceived and/or processed by KAI2 proteins and what structural determinants drive individual KAI2 ligand specificity.

Despite major progress in defining KAI2‐mediated signaling, several fundamental mechanistic and structural questions remain unsolved (Box 2). It is still unclear how KAI2 engages with the F‐box protein MAX2 within the SCF^MAX2^ ubiquitin ligase complex and whether this interaction triggers conformational rearrangements analogous to those observed in SL signaling. Moreover, some plant species encode multiple MAX2 paralogs, raising the possibility that different KAI2 clades or isoforms may exhibit distinct binding preferences or signaling specificities. Furthermore, additional yet unknown interacting partners other than MAX2 or SMAX1/SMXL2 may participate in KAI2‐mediated signaling. Likewise, the mechanism by which SMAX1/SMXL2 co‐repressors are recruited and degraded, and whether they directly bind DNA or act through transcriptional partners (Chang et al., 2025a, 2025b; Wang, Xu, et al., 2020), remains to be fully elucidated. Recent evidence from strigolactone research suggests that SMXLs undergo phase separation to form nuclear condensates that aid in the recruitment of signaling components and possibly ligands, in addition to sequestering transcription factors (Li et al., 2025). Further research into these condensates and liquid–liquid phase separation within the nucleus may reveal that these specialized microenvironments play a key role in VOC perception.

At the structural level, KAI2 clades may differ not only in their ligand specificity but also in the conformational dynamics that underlie their activation, potentially reflecting differences in spatial accessibility, timing, or processivity of their reactions with distinct ligands. Capturing these dynamic transitions will be key to understanding how KAI2s function as both receptors and enzymes. With the rapid advancement of cryo‐electron microscopy and artificial intelligence‐based structural modeling, future studies will resolve the architecture of the KAI2‐MAX2‐SMAX1 complex across functional states and will visualize the molecular rearrangements associated with ligand binding and signal propagation. Integrating these structural insights with biochemical reconstitution, mutagenesis, and functional genomics will be essential for uncovering how KAI2 conformational dynamics and partner selectivity determine signaling outcomes. Ultimately, identifying the authentic/endogenous KL(s), including VOCs and VOC‐derived metabolites, will provide the molecular foundation for connecting volatile perception, receptor activation, and transcriptional regulation in plant adaptive signaling networks.

## CONFLICT OF INTEREST

None of the authors have a conflict of interest to disclose.

## Data Availability

Data sharing not applicable to this article as no datasets were generated or analysed during the current study.
